# Migrant and ethnic minority nurses’ experience of working in European health services: a qualitative study

**DOI:** 10.1590/1980-220X-REEUSP-2022-0104en

**Published:** 2022-09-19

**Authors:** Isabel Antón-Solanas, Beatriz Rodríguez-Roca, Valérie Vanceulebroeck, Nuran Kömürcü, Indrani Kalkan, Isabel Huércanos-Esparza, Antonio Casa-Nova, Nadia Hamam-Alcober, Elena Tambo-Lizalde, Margarida Coelho, Teresa Coelho, Yannic Van Gils, Seda Değirmenci Öz, Arzu Kavala, Enrique Ramón-Arbués, Benjamin A. Jerue, Ana B. Subirón-Valera

**Affiliations:** 1 Universidad de Zaragoza, Facultad de Ciencias de la Salud, Departamento de Fisiatría y Enfermería, Zaragoza, España.; 2 Instituto de Investigación de Aragón, Grupo de Investigación en Enfermería en Atención Primaria de Aragón, Zaragoza, España.; 3 University of Applied Sciences and Arts, Department of Nursing, Antwerpen, Belgium.; 4 İstanbul Aydın Üniversitesi, Sağlık Bilimleri Fakültesi, İstanbul, Türkiye.; 5 İstanbul Medipol Üniversitesi, Sağlık Bilimleri Fakültesi, Beslenme ve Diyetetik Bölümü, İstanbul, Türkiye.; 6 Universidad San Jorge, Faculty of Health Sciences, Villanueva de Gállego, Zaragoza, España.; 7 Instituto Politécnico de Portalegre, Faculdade de Ciências da Saúde, Portalegre, Portugal.; 8 Servicio Aragonés de Salud, Hospital Materno Infantil, Zaragoza, España.; 9 Instituto de Investigación Sanitaria, Hospital Universitario Miguel Servet, Zaragoza, España.; 10 Instituto Politécnico de Portalegre, Escola de Educação e Ciências Sociais, Portalegre, Portugal.; 11 University of Antwerp, Department of Nursing and Midwifery Sciences, Antwerpen, Belgium.; 12 Universidad San Jorge, Facultad de Comunicación y Ciencias Sociales, Zaragoza, España.; 13 Instituto de Investigación de Aragón, Grupo de Investigación Seguridad y Cuidados, Zaragoza, España.; 14 Universidad de Zaragoza, Instituto Universitario de Investigación en Ciencias Ambientales de Aragón, Grupo de Investigación Agua y Salud Ambiental, Zaragoza, España.

**Keywords:** Cultural Competency, Cultural Diversity, Europe, Health Equity, Health Services, Cultural Diversity, Nursing, Qualitative Research, Competência Cultural, Diversidade Cultural, Europa (Continente), Equidade em Saúde, Serviços de Saúde, Diversidade Cultural, Enfermagem, Pesquisa Qualitativa, Competencia Cultural, Diversidad Cultural, Europa (Continente), Equidad en Salud, Servicios de Salud, Diversidad Cultural, Enfermería, Investigación Cualitativa

## Abstract

**Objective:**

To analyze the perception of culture and experience of working in European health services of a purposive sample of qualified migrant and ethnic minority nurses currently living in Belgium, Portugal, Spain and Turkey.

**Method:**

A qualitative phenomenological method was chosen. Individual interviews took place with 8 qualified migrant and ethnic minority nurses currently living in four European countries. Thematic analysis was conducted using Braun and Clark’s stages after qualitative data had been verbatim transcribed, translated into English, and analyzed

**Results:**

Four themes and 4 subthemes emerged from thematic analysis of the transcripts.

**Conclusion:**

Migrant and ethnic minority nurses working in the European Union experience and witness discrimination and prejudice from patients and colleagues due to cultural differences. European health services should closely monitor and address discrimination and prejudice towards migrant and ethnic minority staff and patients, and take initiatives to reduce and, eventually, eradicate them.

## RESUMEN

**Objetivo:**

Analizar la percepción de la cultura y la experiencia de trabajar en los servicios de salud europeos de una muestra intencional de enfermeros calificados de minorías étnicas y migrantes que actualmente viven en Bélgica, Portugal, España y Turquía.

**Método:**

Se decidió utilizar un método cualitativo fenomenológico. Se realizaron entrevistas individuales con 8 enfermeros calificados inmigrantes y de minorías étnicas que actualmente viven en cuatro países europeos. El análisis temático se realizó utilizando las etapas de Braun y Clark después de que los datos cualitativos fueran transcritos palabra por palabra, traducidos al inglés y analizados.

**Resultados:**

Del análisis temático de las transcripciones surgieron 4 temas y 4 subtemas.

**Conclusión:**

Los enfermeros de minorías étnicas y migrantes que trabajan en la Unión Europea experimentan y son testigos de la discriminación y de los prejuicios de los pacientes y colegas sobre la base de la diferencia cultural. Los servicios de salud europeos deben monitorear de cerca y abordar la discriminación y los prejuicios hacia el personal y los pacientes de minorías étnicas y migrantes, y tomar iniciativas para reducirlos y, eventualmente, erradicarlos.

## RESUMO

**Objetivo:**

Analisar a percepção da cultura e experiência de trabalho em serviços de saúde europeus de uma amostra intencional de enfermeiros qualificados migrantes e de minorias étnicas que moram atualmente na Bélgica, Portugal, Espanha e Turquia.

**Método:**

Optou-se pelo método fenomenológico qualitativo. Realizaram-se entrevistas individuais com 8 enfermeiros qualificados migrantes e de minorias étnicas que moram atualmente em quatro países europeus. A análise temática foi realizada por meio das etapas de Braun e Clark após os dados qualitativos terem sido transcritos na íntegra, traduzidos para o inglês e analisados.

**Resultados:**

Quatro temas e 4 subtemas emergiram da análise temática das transcrições.

**Conclusão:**

Enfermeiros migrantes e de minorias étnicas que trabalham na União Europeia vivenciam e testemunham discriminação e preconceito de pacientes e colegas devido a diferenças culturais. Os serviços de saúde europeus devem acompanhar de perto e combater a discriminação e o preconceito contra os trabalhadores e pacientes migrantes e de minorias étnicas, e tomar iniciativas para os reduzir e, em seguida, erradicá-los.

## INTRODUCTION

The European Union’s (EU) social policy aims “to promote employment, improve living and working conditions, provide an appropriate level of social protection and develop measures to combat exclusion”^(1)^. Nevertheless, social exclusion and inequality have grown to be serious issues in the European society during the last few years. This problem was addressed by the initiative, which was sponsored by the Erasmus + program under Key Action 203 Strategic Partnerships for Higher Education. It is an example of collaboration between 4 European high education institutions. In this paper, we present the results from our investigation of qualified migrant and ethnic minority nurses’ perception of culture and experience of working in European healthcare services.

Demographic trends predict a rapid growth in racial and ethnic minority populations worldwide^(2)^. The EU population is projected to increase from 446.8 million in 2019 to 449.3 million in 2026^(3)^. According to recent estimates of the International Labour Organization, there are 164 million migrant workers worldwide, nearly one fourth of whom are located in North America as well as Northern and Western Europe^(4)^.

An increasingly multicultural population is presenting European health organizations with the challenge of delivering care to patients with diverse healthcare beliefs, languages and practices^(5)^. In addition, whilst the social demand for care is increasing due to longer life expectancy and a raise of multimorbidity and chronic diseases^(6)^, this growing demand is being confronted with declining supply of qualified nurses in developed countries^(7)^. Thus, health systems have sought ways to increase the number and diversity of nursing staff available. Many developed countries, in addition to the strategy to educate and train domestic healthcare professionals, have promoted the immigration of foreign-trained nurses^(8)^. Specifically, within the Organisation for Economic Co-operation and Development (OECD) countries, the number of foreign-trained nurses increased by 20% over the five-year period from 2011 to 2016 (to reach nearly 550.000)^(8)^. Yet, although the population of registered nurses is growing in diversity, migrant and ethnic minority (MEM) nurses remain underrepresented^(2)^.

The migration of nurses into Spain, Belgium, Portugal and Turkey has increased in the past few years, but the composition and configuration of the nursing workforce varies from one nation to another. Data are not consistent but, according to the OECD Health Statistics 2021^(9)^, the annual inflow of foreign-trained nurses in Spain was 617 in 2020, representing about 0.2% of the nursing workforce^(10)^. In Belgium, the percentage of foreign-trained nurses was 4.11% of the total nursing workforce in the same year^(9)^. In Portugal, the latest percentage of foreign-trained nursing personnel is from 2014 and it amounted to 1.82% of the nursing workforce, whilst in Turkey it amounted to 0.3% in 2015^(9)^.

Nurse leaders, leading nursing organizations and other stakeholders have articulated the need for more diversity in nursing^(2)^. Evidence suggests that a culturally diverse nursing workforce is crucial to meet the needs of increasingly diverse populations and provide patient-centered, culturally competent nursing care, improves access to health services, and reduces health disparities^(11)^. Furthermore, a diverse nursing workforce is especially important since nurses make up the most numerous groups of healthcare providers and are in close contact with all patients, including those who are culturally diverse^(5)^.

Prior studies on the work experiences of MEM nurses have found differences in job satisfaction and have called for further research into the factors underlying these differences^(5)^. In addition, recent studies have presented disturbing findings on the cultural bias and discrimination faced by MEM nurses in countries with a long tradition of cultural diversity and migration, such as Japan^(12)^, the US, the UK^(13)^, and Australia^(14)^. However, less is known about the work experiences of MEM nurses in other European nations with a more recent history of recruitment of foreign-trained nurses.

Analyzing the work experiences of MEM nurses in Spain, Belgium, Portugal and Turkey may contribute to the understanding of their unique experiences, as well as the difficulties and challenges they encounter. In addition, it may help nursing and healthcare leaders and decision-makers identify strategies to improve the MEM nurses’ experiences and promote recruitment and retainment of more MEM in their healthcare systems, thus enhancing cultural competency and reducing health inequalities^(5)^. Therefore, the aim of this study was to examine the work experiences of migrant and qualified MEM nurses working in healthcare services across Spain, Portugal, Belgium, and Turkey.

For the purpose of this study, a MEM nurse is defined as a locally-born nurse who belongs to a cultural minority clearly distinct from the large cultural majority, such Gypsy Roma Travelers, or a “foreign-born nurse who has migrated to work in a healthcare setting in another country where that nurse’s race and/or ethnicity are not prominent constituents of the whole”.

## METHOD

### Design

As the research aims to elicit the experiences of MEM qualified nurses working in healthcare services across Spain, Portugal, Belgium, and Turkey, a phenomenological approach has been selected. This is appropriate as phenomenological research allows researchers to understand complex phenomena through the participants’ lived experience, meaning, and perspectives^(15)^.

The COREQ reporting guidelines were used in both the framing and reporting of this study to guarantee that sufficient details on the methods of data collection, analysis, and interpretation were provided^(16)^.

### Participants and Study Location

The study target population consisted of 8 qualified nurses with a MEM background working in Spanish, Portuguese, Belgian, or Turkish health services, both public and private, to broaden the participants’ experience of the phenomenon. We used a convenience sampling technique, supported by clearly defined selection criteria. Inclusion criteria for taking part in the study included: 1) qualified nurses with a MEM background employed by a local healthcare provider, 2) individuals with at least two-years of post-qualifying experience working in Spanish, Belgian, Turkish or Portuguese health services, 3) individuals who agreed to the conditions of the study and gave informed consent to participate. Participants with less than one-year experience working in their current service were excluded.

The participants’ line managers and/or nurse coordinators/directors from each service served as the gatekeepers who provided access to the workers. A formal invitation to take part in this study was sent via institutional email to the potential participants. None of the candidates who were invited to participate refused to take part in this investigation.

### Data Collection

The participants were individually interviewed in a secure and unbiased setting. A scholar from each of the study sites who is skilled in qualitative data gathering techniques conducted the interviews. Although, originally, we planned for interviews to take place in person, some were made online due to the circumstances arising from the COVID-19 pandemic.

**Chart 1:**
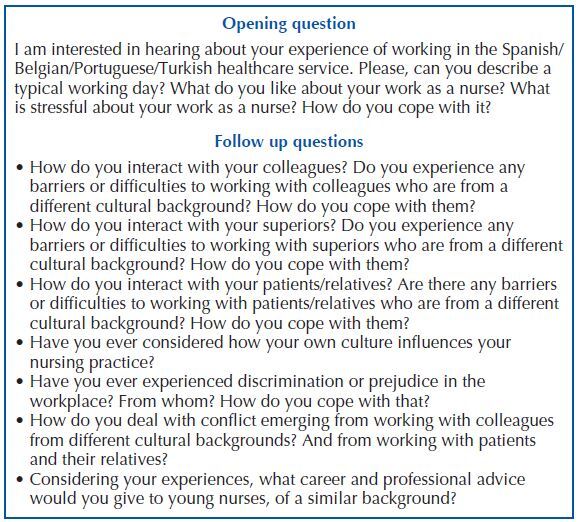
Topic guide for semi-structured interviews with qualified nurses from MEM backgrounds – Zaragoza, Spain, 2020.

Interviews were verbatim captured on audio, then transcribed. Academics who conducted the interview and are fluent in English then translated the results into English. The identical interviewing protocol, created by the primary investigator (IA-S) and approved by the research team, was used by all researchers (see Chart 1).

Additionally, participants were requested to complete a sociodemographic survey to describe the features of our sample, including the following variables: age (years), sex, marital status, race/ethnicity, religious affiliation, socioeconomic level, country of birth, country of work, professional experience (years), number of languages spoken, cultural competence training, involvement with diverse patients/organizations, and experience working with patients from diverse cultural backgrounds.

### Data Analysis

The sociodemographic data was analysed using descriptive statistics, which used frequency and percentage for qualitative variables and mean and standard deviation for quantitative ones.

Anonymized transcripts underwent a qualitative thematic analysis that included familiarising oneself with the data, creating preliminary codes, looking for themes, examining themes, defining and labelling themes, and producing the report.

**Chart 2:**
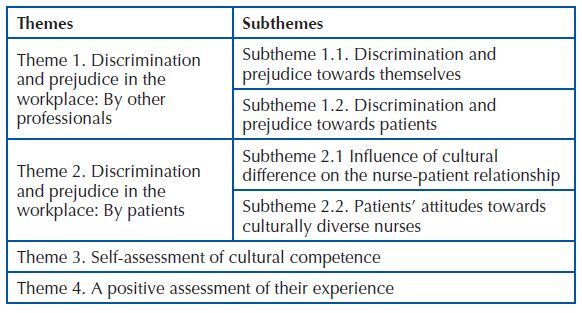
Themes and subthemes – Zaragoza, Spain, 2020.

Separately, two researchers (ABS-V; BR-R) manually analyzed the transcripts and identified overarching themes and subthemes from the information. Thematic analysis of the transcripts revealed four themes and twelve subthemes (Chart 2).

### Ethical Considerations

Before the study began, it was approved by the Autonomous Communities of Aragon’s Ethics Committee (C.P. – C.I. PI20/097; 1^st^ April 2020). The participants’ employers were also asked for consent before any data were gathered. Following a brief description of the study including information about their right to withdraw participation at any moment during the process without having any impact on their professional career, all participants willingly agreed to take part in the study protocols. Prior to the interviews, each participant volunteered their informed consent. Compliance with the General Data Protection Regulation was ensured at all times, as well as participant anonymity and confidentiality (RGPD 2016/679).

## RESULTS

Table 1 lists the sample’s sociodemographic and cultural characteristics. The participants were 29,6 on average. Nearly 90% of the participants were female. Five participants were single, while three were either married or in committed relationships. The bulk of the interviewees identified themselves as coming from a low social class (75%) and having an ethnic background (75%). Most of the participants were Christian (50%), Muslim (37.5%), or Jew (12.5%). Their countries of birth were varied, including Cape Verde, Morocco, Russia, Romania, Macedonia, and Algeria. The majority of the participants belonged to a culturally diverse family and/or group of friends and had experience working with culturally diverse patients. Only 2 out of the 6 had some previous cultural competence training.

**Table 1.** Sociodemographic and cultural characteristics of the sample (n = 8) – Zaragoza, Spain, 2020.

Variables	Frequency (%) or Mean (SD)
Age (years)	29.60 (8.08)
Clinical work experience (years)	8.67 (7.11)
Sex	1 (12.5%)
Male	7 (87.5%)
Female	
Marital status	5 (62.5%)
Single	3 (37.5%)
Married/partner	
Race/ethnicity	6 (75%)
White	2 (25%)
Black	
Religious affiliation	3 (37.5%)
Catholicism	1 (12.5%)
Protestantism	3 (37.5%)
Islamism	1 (12.5%)
Judaism	
Adherence to religion	6 (75%)
Practicing	2 (25%)
Non-practicing	
Socioeconomic level	2 (25%)
Middle social class	6 (75%)
Low social class	
Country of birth	2 (25%)
Cape Verde	1 (12.5%)
Macedonia	2 (25%)
Morocco	1 (12.5%)
Romania	1 (12.5%)
Russia	1 (12.5%)
Algeria	
Country of work	2 (25%)
Belgium	2 (25%)
Portugal	3 (37.5%)
Spain	1 (12.5%)
Turkey	
Number of languages spoken	1 (12.5%)
One	7 (87.5%)
More than one	
Belonging to a culturally diverse family	7 (87.5%)
Yes	1 (12.5%)
No	
Prior cultural competence training	2 (25%)
Yes	6 (75%)
No	
Prior/current voluntary work with patients from diverse cultural backgrounds and organizations (i.e. NGOs, etc.)	3 (37.5%)
Yes	5 (62.5%)
No	
Experience in caring for patients from diverse cultural backgrounds	6 (75%)
Yes	2 (25%)
No	

### Theme 1. Discrimination and Prejudice in the Workplace: By Other Professionals

This theme integrates the participants’ descriptions of discrimination, prejudice, and racism in the workplace; sometimes these negative attitudes were directed towards themselves, and other times they were directed towards their patients.

#### Subtheme 1.1 Discrimination and Prejudice Towards Themselves

It is worth noting that the participants’ descriptions of interpersonal relationships with their colleagues were varied. Not surprisingly, their comments became more negative as the interview progressed, with more participants describing discriminatory behaviors and prejudice experienced in the workplace.

*I have no difficulty for everything that has been my way of life. I’ve always been very integrated in a very different team* (N1, Cape Verde, female, works in Portugal)

*I have already felt prejudice to myself, to my colleagues and to other professional groups, including assistants and doctors* (N1, Cape Verde, female, works in Portugal)

Often, the participants perceived that prejudiced and discriminatory comments and behaviors were expressed in a subtle and covert way; *“behind their backs”.*

*Colleagues also didn’t say things right to my face, but they did behind my back. The hardest thing for me was to find an appropriate way to work with colleagues, both day-to-day and during briefings* (N3, Russian, female, works in Belgium)

One of the nurses working in Belgium identified (lack of) language proficiency with discriminatory attitudes and behaviors:

*Other Ukrainian colleagues of mine who are less proficient in the language are not treated as well by fellow nurses. I was very lucky because I grew up in a Belgian family, so I have a good knowledge of Dutch. There are many foreign nurses who are not treated well. When they do get treated well, they are usually nurses who have a good command of the language* (N3, Russian, female, works in Belgium)

More often than not, discriminatory attitudes and behaviors came from cultural majority colleagues. It was rare for line managers and superiors to be involved in cultural conflict.

*I don’t perceive any barriers between my line manager and me* (N6, Romanian, female, works in Spain)

*I did not and do not have any problems with my superiors either* (N5, Macedonian, female, works in Turkey)

#### Subtheme 1.2. Discrimination and Prejudice Towards Patients

Despite denying experiencing and witnessing them earlier on in the interview, sometimes the participants expressed their disgust over discriminatory behaviors and attitudes in the workplace, not only against themselves, but also against patients.

*I don’t want to complain about my colleagues, but racism is increasing, even in the hospital. Some nurses do their best, but others put a sticker on patients´ heads as soon as they read that they are of a different origin* (N3, Russian, female, works in Belgium)

They were deeply moved when they witnessed discriminatory or prejudiced behaviors against patients in the workplace. Often, racist comments were expressed by other healthcare professionals against culturally diverse patients, even children. The participants had no difficulty detecting and describing examples of racism in the workplace and demonstrated more empathy than their cultural majority colleagues.

*The longer I work, the more racism occurs. I hear many unjustified comments about Jewish patients. Patients see me as more understanding than my other colleagues* (N3, Russian, female, works in Belgium)

*Once we had a Rumanian child in the boxe and I heard someone say: ‘you are invading us’, or something like that so yes, I feel like…, yes, maybe they do it unconsciously, but I don’t know, it’s out of order* (N6, Romanian, female, works in Spain)

When faced with these situations, the participants’ responses were varied. Sometimes, they chose not to intervene:

*In those cases, in which I witness an action that I think is racist, I usually avoid confrontation* (N7, Algerian, male, works in Spain)

But in some cases, they expressed their anger and verbally challenged the person whose attitude was prejudiced, discriminatory, or racist:

*I usually don’t confront them, but sometimes I have said something because…, they just shouldn’t say that* (N6, Romanian, female, works in Spain)

*Sometimes I don’t say anything, sometimes I do. They have a right to be here, these people work, like you and me, they have a right to health care and so do their children* (N6, Romanian, female, works in Spain)

### Theme 2. Discrimination and Prejudice in the Workplace: By Patients

Negative attitudes towards culturally diverse nurses came not only from work colleagues but also from patients. This theme describes some aspects of the nurses’ interpersonal relationship with patients who belonged to a different cultural background.

*In my workplace, I only felt this differentiation on the part of the patients* (N2, Cape Verde, female, works in Portugal)

#### Subtheme 2.1 Influence of Cultural Difference on the Nurse-Patient Relationship

The participants believed that cultural differences should not affect the nurse-patient relationship. However, this was not always the case. One of the nurses working in Spain said that she hoped to be judged not by her cultural identity but by her professionalism and the quality of her work.

*I don’t know. I hope not. I hope they don’t judge me for my cultural origin* (N6, Romanian, female, works in Spain)

*There are regularly Belgians who ask where I am from. They are very sympathetic, but I feel that they are distrustful. I also notice that they deal better with white nurses* (N3, Russian, female, works in Belgium)

#### Subtheme 2.2 Patients’ Attitudes Towards Culturally Diverse Nurses

The participants described a range of different patient attitudes towards them, including interest or curiosity…

*Well, perhaps it’s because I am a Muslim nurse and I wear a scarf (…), there is always a patient who’s curious about the fact that I wear it, or what it means for me. (…) They look fixedly at me and that sometimes makes me uncomfortable (…). They never say anything to me but sometimes I feel observed”* (N8, Moroccan, female, works in Spain)

…but also, racism and stereotype:

*“I have known situations where the patient did not want to be cared for by an African nurse. That was going too far for me. Whenever such situations arise, I try to intervene* (N3, Russian, female, works in Belgium)

*In the interaction with users I have already felt some barriers because I am of another color. Some users refused to be treated for the simple fact that I am black* (N2, Cape Verde, female, works in Portugal)

### Theme 3. Self-Assessment of Cultural Competence

The participants believed that they were more empathetic towards culturally diverse patients than their cultural majority colleagues; this was sometimes expressed directly and sometimes it was implicit in their discourse:

*Perhaps I’m more empathetic than other people, it’s possible, because I’m not from here, I’m on my own and, I don’t know, maybe I am more empathetic and I understand people a bit better* (N6, Romanian, female, works in Spain)

Interestingly, the participants saw cultural difference as exerting a positive influence on the nurse-patient relationship and thus, as potentially improving the quality of their nursing practice.

*My culture helps me to provide better care because I grew up as a Muslim and I’m also open to other cultures* (N4, Moroccan, female, works in Belgium)

### Theme 4. A Positive Assessment of their Experience

Finally, the participants generally expressed great satisfaction with their work and a vocation for nursing, emphasizing the relationship with their patients and highlighting positive aspects of their work and working with their colleagues.

*I get a lot of satisfaction from the work itself. Counseling patients on things they can’t do themselves, but also having social contact with the patients* (N3, Russian, female, works in Belgium)

*Working in the neonatal unit is quite stressful, when you have to run to a delivery, when it’s complicated, when the child is unwell. I don’t know, we work as a team then and we support each other* (N6, Romanian, female, works in Spain)

## DISCUSSION

Nursing continues to be a female-dominated profession^(17)^, as reflected in the sex characteristics of our sample. Our participants came from a variety of countries and ethnic backgrounds, reflecting Europe’s diversity and growing rates of immigration in virtually all member states^(18)^. The majority of our participants classified themselves as being from a low social class. This may be a true reflection of their socioeconomic status, but it is possible that our participants’ perception of their socioeconomic level was shaped by their cultural background^(19)^. Although only a minority of participants had had prior training in cultural competence, most of them belonged to a culturally diverse family and/or group of friends and spoke more than one language, characteristics which have previously been associated with a higher level of cultural competence^(20)^.

We observed that, at first, the participants were reluctant to express any cultural difficulties arising in the workplace. However, as the interview progressed, MEM nurses described many examples of discrimination and prejudice towards themselves, as well as towards other culturally diverse colleagues and patients, usually on the basis of nationality, race and ethnicity, but also of language proficiency and religion. According to previous studies^(15)^, migrant and minority nurses often report discrimination and racism at work. Our participants experienced cultural discrimination in contact with not only patients and relatives, but also other nurses and healthcare professionals. Some of them described these episodes as isolated incidents. However, cultural discrimination in the healthcare environment occurs more often than may be expected^(21)^. According to a recent survey on diversity issues carried out in the UK, 63% of nurses had observed racial discrimination or disadvantage affecting someone else other than themselves in the previous year^(22)^. This is important as the health of MEM nurses is affected by experiences of discrimination and prejudice at work^(23)^. Further, it affects staff morale, resulting in high turnover rates^(24)^. Health services should carefully monitor and address these incidents^(25)^, implementing a zero-tolerance approach to discrimination and abuse in the workplace, as well as taking initiatives to reduce and, eventually, eradicate discrimination and prejudice on the basis of racial, ethnic, or other difference. This is supported by the Racial Equality Directive (RED) adopted in 2000 by The Council of the EU, which states that discrimination based on racial and ethnic origin is prohibited in the EU.

The MEM nurses described examples of racist comments expressed by other healthcare professionals towards culturally diverse patients, including children. They were deeply moved when they witnessed discriminatory or prejudiced behaviors towards patients in the workplace. Their responses to these episodes were varied; whilst some chose not to intervene, others verbally challenged the abuser. It is unclear whether our participants reported these incidents to their line managers. Some authors^(26)^ have argued that MEM nurses may lack the confidence to report them for fear of isolation and retaliation, which is understandable to some extent, but also extremely disturbing. According to the Workforce race inequalities and inclusion in NHS providers’ report^(27)^, ensuring psychologically (and professionally) safe routes for raising concerns through the appointment of Freedom to Speak Up Guardians, as well as the implementation of other complementary interventions like establishing minority staff networks and developing routes for staff to raise concerns, contribute to creating a safer atmosphere for healthcare professionals to raise concerns.

Despite not having had any prior training in cultural competence (in their majority), our participants’ self-assessment of cultural competence was good. In their view, their ability to empathize with culturally diverse patients was greater than that of their cultural majority peers. They attributed this ability to specific cultural features, such as their religion and religious values, and also the fact that, as migrants and/or culturally diverse individuals themselves, they were able to “put themselves in their patients’ shoes” more easily. Numerous voices have claimed for a more diverse nursing workforce to provide quality, culturally competent patient care, improve culturally diverse patient outcomes, and reduce health disparities^(2)^. Many countries around the world, including the UK and the US, have recognized this need and are implementing nation-wide initiatives to close the diversity gap within nursing. However, more work needs to be done to ensure that the nursing workforce reflects the rich and growing diversity of the European population^(12)^. A top-down, structural, and systematic approach is needed to address homogeneity in the nursing workforce, starting with leaders and governing bodies, by recruiting and promoting MEM individuals to senior positions in the healthcare, educational and research workforce, cultural competency training for current leaders and staff in both managerial and clinical roles, increasing recruitment and retention of both student and qualified nurses, and integrating cultural competence in nursing education.

Discrimination and racial harassment have a significant negative impact on job satisfaction and are associated with unhealthy behaviors, such as smoking, psychological distress^(28)^, and negative job outcomes such as sickness absence, leading some nurses to quit their profession. Yet, despite having both experienced and witnessed discrimination and prejudice in the workplace on the basis of cultural difference, most of the MEM nurses finished the interview on a positive note, expressing their satisfaction with their job and highlighting positive aspects such as the opportunity to work as a team. Some authors^(29)^ have argued that the impact of discrimination and prejudice in the workplace for cultural reasons tends to diminish over time. A recent study^(30)^ carried out in Singapore found a positive association between acculturation and quality of life on a sample of international nurses. However, acculturation can be a long and complex process, which should be overseen and supported by healthcare services. Most of our participants had lived in their current countries of residence for a long time; some, like the two nurses working in Belgium, had even been raised there. Thus, it is likely that they had become acculturated into their respective societies and healthcare services by the time they were interviewed.

We would like to highlight some limitations of this study and offer recommendations for further investigation. Firstly, it was not our purpose to present a representative description of MEM nurses in Europe and, therefore, the characteristics of our sample may not be applicable to other MEM nursing populations. Secondly, participant recruitment and data collection in Portugal, Belgium, and Turkey was affected by the COVID-19 crisis, and the number of MEM nurses working in these countries was smaller than initially planned. This may have affected the depth and quality of the information and, thus, limited the interpretation of the findings. Thirdly, although we analysed the MEM nurses’ testimonies as a whole, we acknowledge that they are not a homogenous population and that their perceptions and experiences were influenced by a wide range of factors, including the culture of each separate health service, the population’s health needs, the characteristics of most of the population in each country and their social support, to mention but a few. Finally, most of the MEM nurses had been living in their respective countries of residence for a long time. Future studies should investigate the experiences of newly arrived migrant nurses.

Subheadings may be used to split this section. It should give a clear and succinct explanation of the experimental findings, their interpretation, and any possible experimentalinferences.

## CONCLUSION

MEM nurses working in European health services experience discrimination and prejudice from patients and colleagues mainly on the basis of nationality, race and ethnicity, but also of language proficiency and religion. In addition, MEM nurses witnessed racist behavior and attitudes towards culturally diverse patients. However, it was unclear whether these episodes were reported to their line managers or other individuals within their respective health services. Despite not having had any previous cultural competence training, the MEM nurses’ self-assessment of cultural competence was good. They attributed this assessment to specific cultural features, such as their religion and religious values, and their enhanced ability to empathize with culturally diverse patients.

Understanding MEM nurses’ experience of working in European health services will help lead nurses, health service managers, and policy and decision-makers effectively plan and implement strategies to improve job satisfaction and increase diversity in the nursing workforce. Findings from this study suggest that European health services should closely monitor and address discrimination and prejudice on the basis of cultural difference towards MEM staff and patients, and take initiatives to reduce and, eventually, eradicate them. Our results indicate that MEM nurses were generally satisfied with their job. However, this may be due to the fact that they were already acculturated to their host societies and health services. However, acculturation can be a long and complex process. European health services should oversee the process of acculturation of new MEM nursing staff and offer support to facilitate their transition.
